# Triglyceride-Glucose Index Correlate With Telomere Length in Healthy Adults From the National Health and Nutrition Examination Survey

**DOI:** 10.3389/fendo.2022.844073

**Published:** 2022-06-02

**Authors:** Lihua Hu, Qiaojian Zhang, Yi Bai, Guiping Hu, Jianping Li

**Affiliations:** ^1^ Department of Cardiology, Peking University First Hospital, Beijing, China; ^2^ Department of Occupational and Environmental Health Sciences, School of Public Health, Peking University, Beijing, China; ^3^ Department of Epidemiology and Health Statistics, Peking University, Beijing, China; ^4^ School of Engineering Medicine, Beihang University, Beijing, China; ^5^ Beijing Advanced Innovation Center for Big Data-Based Precision Medicine, and Advanced Innovation Centre for Biomedical Engineering, Beihang University, Beijing, China

**Keywords:** insulin resistance, triglyceride glucose index, homeostatic model assessment of insulin resistance, leukocyte telomere length, threshold effect

## Abstract

**Aim:**

The present investigation was designed to test the association between leukocyte telomere length (LTL) and two simple markers of insulin resistance, that is, homeostatic model assessment of insulin resistance (HOMA-IR) and triglyceride-glucose (TyG) index in U.S. adults without metabolic diseases.

**Methods:**

A total of 6489 U.S. adults without diabetes from NHANES 1999–2002 were analyzed. TyG index was calculated as ln [fasting triglycerides (mg/dL) × fasting glucose (mg/dL)/2]. HOMA-Index was calculated as fasting plasma glucose (mmol/L) × fasting serum insulin (mU/mL)/22.5. LTL was obtained using the quantitative polymerase chain reaction method. Multivariate linear regression analysis was assessed to evaluate the association of TyG index HOMA-IR with LTL. We further conducted a generalized additive model (GAM) and a fitted smoothing curve with penalized spline method.

**Results:**

It was found that the mean LTL was 5796.1 bp in the measured healthy adults. Overall, TyG index was significantly associated with LTL, while HOMA-IR was not. Compared with participants in tertile 1 of the TyG index, the β (95% CI) for those in the second (8.27 to 8.77) and third (≥ 8.77) were -4.31 (95% CI: -48.12~39.49) and -95.98 (95% CI: -145.08~-46.89), respectively. Subjects with TyG index ≥ 8.77 had statistically significant shorter LTL (β = -93.33, 95%CI: -134.33~-52.32), compared with TyG index < 8.77. We further explored a dose-response relation between TyG index by a decile approach [≤ 7.81 (reference), 7.81-8.04, 8.04-8.21, 8.21-8.37, 8.37-8.52, 8.52-8.68, 8.68-8.83, 8.83-9.03, 9.03-9.33, and >9.33] and LTL. Five subgroups (TyG index 7.81-8.04, 8.04-8.21, 8.21-8.37, 8.37-8.52, and 8.52-8.68) did not show significant effect on LTL; while there was a significantly shorter LTL for participants with the TyG index > 8.68, supporting a threshold effect of TyG index on LTL.

**Conclusions:**

The results suggested that higher TyG index (> 8.68) was closely related to shorter LTL and the TyG index was better associated with LTL than HOMA-IR.

## Introduction

The widespread incidence of metabolic diseases has risen sharply during the last decades, and prevalence portends an even greater growth in the future, which has brought great threat or damage to the life quality of patients, and a burden to society and economy (1, 2). Other nontraditional risk factors, such as calorie consumption and a sedentary lifestyle, are undoubtedly substantial contributions to this rise (3–5). Therefore, clinical medicine and basic researchers are highly concerned and have deeply studied them from different aspects.

Insulin resistance (IR) is one of the main manifestations of most metabolic diseases (e.g., metabolic syndrome, obesity, impaired glucose tolerance, and diabetes), and associated with inflammation and oxidative stress (6). IR is defined as a condition in which normal insulin concentrations elicit a less-than-normal physiologic response (7, 8). Reduced insulin sensitivity leads to lower insulin-stimulated glucose absorption as well as higher plasma glucose and triglycerides (TG). Previous studies showed that IR might reflect the pathophysiological process of metabolic and inflammatory status at the time of sample collection (8).

Given that IR is a critical hallmark of diabetes and most other metabolic illnesses, as well as the fact that IR is a key component of current preventative methods, the homeostatic model assessment of IR (HOMA-IR), “gold standard” of IR, has not been widely used in the clinic due to the complex and expensive process (9). Recently, the triglyceride-glucose (TyG) index, calculated as ln[TG (mg/dL) × fasting glucose (mg/dL)/2], was proposed as a reliable and inexpensive surrogate biomarker to reflect IR (10). In both non-diabetic and diabetic individuals, the TyG index has been shown to be highly linked with IR and to perform better than the homeostasis model in assessing IR (11). Studies have shown that the TyG index is associated with an increased risk of diabetes, hypertension, and nonalcoholic fatty liver disease, and might predict the development of cardiovascular events (10–12). It was confirmed that the TyG index was more sensitive and specific than the steady-state equilibrium model and hyperinsulinemic euglycemic clamp test in evaluating insulin resistance index, and it was also a sensitive index for early prediction of individual diabetes risk (10, 12). With the heightened IR, the risk of aggregation of metabolic risk factors increases gradually (13, 14). Furthermore, with the increasing concern regarding the disturbances of glucose and lipid homeostasis in the general population, the prevalence of coexistence of abnormal glycolipid metabolism IR is also increasing year by year. In this sense, studies that focus on the TYG index are urgently needed.

Other than hormones and metabolic markers, leukocyte telomere length (LTL), the biomarkers of replicative cell aging, have been found to reflect biological age and blood vessel oxidative stress in studies (15, 16). Telomeres are the TTAGGG repeating sequences at the ends of linear chromosomes that protect chromosomal integrity during cell division from degradation (17). LTL appears to be a biomarker that captures the lifetime burden of oxidative stress and inflammation, according to the researchers (18, 19). People with short LTL may be prone to develop accelerated vascular aging (19, 20), atherosclerosis (21), hypertension (22), and type 2 diabetes mellitus (23). However, the function of telomere biology in arterial stiffness is still unknown, as is the existence of shared pathophysiological pathways linking arterial aging and replicative cellular senescence (23). Few studies, however, have reported the association between LTL and the biomarkers of pathophysiological process for metabolic and inflammatory status. Al-Attas et al. found LTL was in relation to IR, inflammation, and obesity among Arab youth (24). There is little information available about the relationship between LTL in relation to metabolic diseases risk factors such as the TyG index.

Many agencies (e.g., U.S. National Institutes of Health (25), the Canadian Diabetes Association (26)) underline the need to understand the links among risk factors before the formation of metabolic diseases with the aim of informing future prevention and research strategies. Whereas the TyG index is a simple new synthetic and feasible biomarker that has been proven to be of better value than previous indexes in reflecting IR and predicting metabolic diseases. Therefore, the purpose of this study was to explore the association of two simple markers of IR, that is, HOMA-IR and the TyG index with LTL in U.S. adults without metabolic diseases such as diabetes, using data from the National Health and Nutrition Examination Survey (NHANES) and to test which of the two was the best predictor of LTL.

## Methods

### Study Population

NHANES was an ongoing cross-sectional study done by the Centers for Disease Control and Prevention (CDC) to measure the health and nutritional status of the civilian, non-institutionalized US population. The NHANES study protocols were approved by the National Center for Health Statistics (NCHS) Ethics Review Board. All study participants signed written informed consent forms. The NHANES datasets are available on DataDryad (https://doi.org/10.5061/dryad.d5h62).

**Figure 1:**
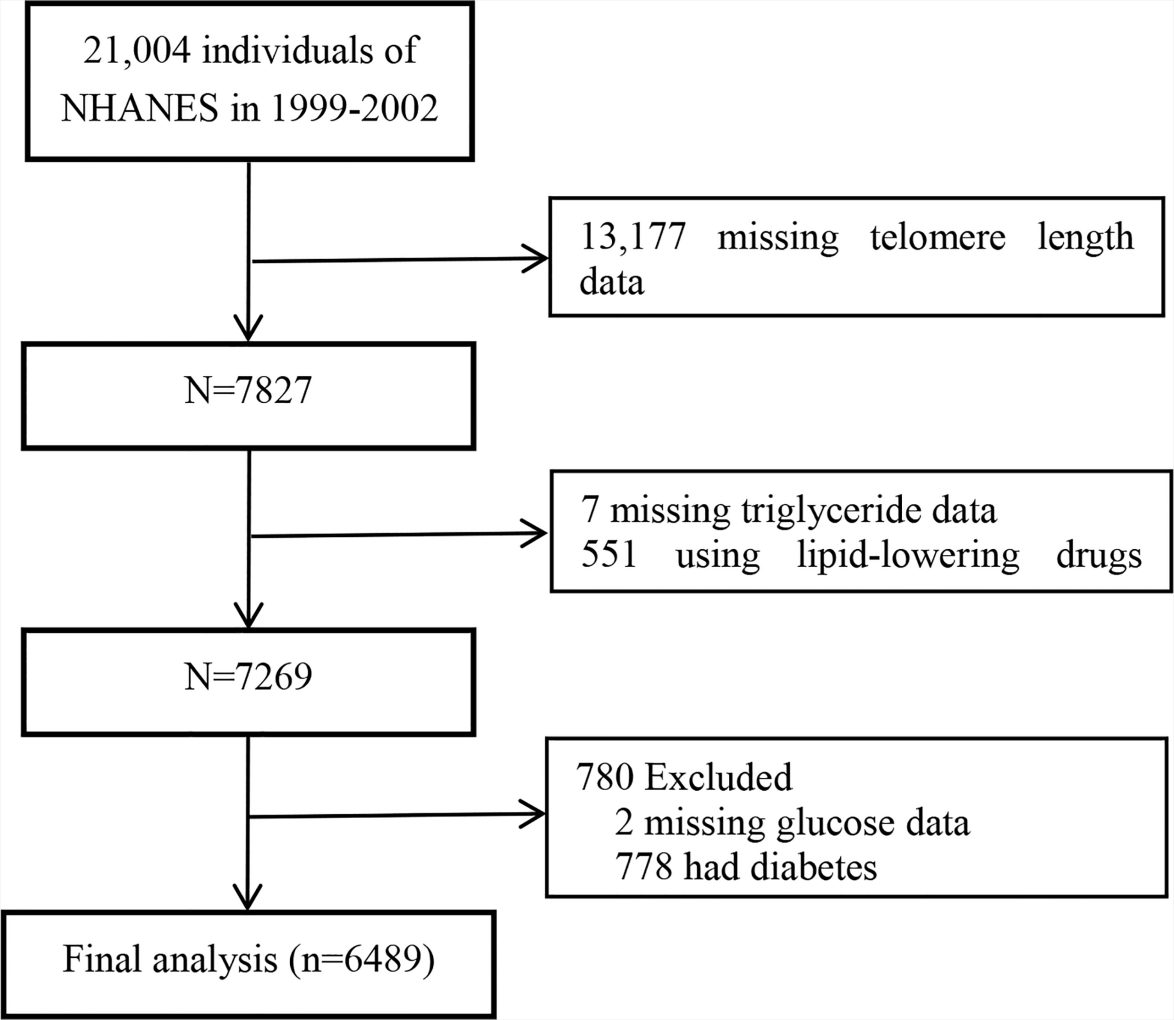
Flow chart of study participants.

Data from the 1999–2000 and 2001–2002 cycles of NHANES were pooled because of available data of LTL in these two data collection cycles. In total, 7827 participants with LTL data were included. Participants with missing triglyceride data (n=7), glucose data (n=2), or using lipid-lowering drugs (n=551) were excluded. Considering the possibility of IR bias among the population with diabetes, we further excluded participants with diabetes (n=778). Finally, a total of 6489 participants were included in this study. The selection process of the study analytic sample is detailed in **Figure 1**.

### Data Collection

All information was collected by trained researchers. Data included demographics characteristics (sex, age, race/ethnicity, poverty to income ratio), health-related behavior (smoking, alcohol consumption, physical activity), education, history of diseases (hypertension, diabetes), using medications, anthropometric measurements (height, weight, waist circumference, blood pressure) as well as laboratory data [fasting blood glucose (FBG), total cholesterol (TC), TG, high-density lipoprotein-cholesterol (HDL-C]), serum uric acid (SUA), serum creatinine, and C-reactive protein (CRP)]. Fasting venous blood samples were collected. Laboratory data were determined using automatic clinical analyzers. The details of the covariate measurement process are available at http://cdc.gov/nchs/nhanes.

The following formula was used to compute the body mass index (BMI): BMI=weight (kg)/height (m^2^). Hypertension was defined as a mean systolic blood pressure (SBP) ≥140 mmHg or a mean diastolic blood pressure (DBP) ≥90 mmHg or a positive response to the questions: “Have you been told on two or more different visits that you had hypertension?” or “Are you taking prescribed medicine to lower blood pressure” (27). Diabetes was defined as a fasting glucose concentration >126 mg/dL or a self-reported medical diagnosis of diabetes. Estimated glomerular filtration rate (eGFR) was calculated *via* using the newly developed Chronic Kidney Disease Epidemiology Collaboration equation (28): eGFR = 141 × min(Scr/κ, 1)α × max(Scr/κ, 1)-1.209 × 0.993Age × 1.018 [if woman] × 1.159 [if black], where Scr is serum creatinine, κ is 0.7 for women and 0.9 for men, α is -0.329 for women and -0.411 for men, min indicates the minimum of Scr/κ or 1, and max indicates the maximum of Scr/κ or 1.

### Exposure Variable and Outcomes

In our study, the exposure variable was the TyG index and HOMA-IR. The TyG index was calculated by the formula ln [fasting TG (mg/dL) ×fasting glucose (mg/dL)/2] (29). TyG index was divided into 3 tertiles: <8.27 (tertile 1), 8.27-8.77 (tertile 2), and ≥8.77 (tertile 3). HOMA-index was calculated as fasting plasma glucose (mmol/L) × fasting serum insulin (mU/mL)/22.5. The outcome variable was LTL. Aliquots of purified DNA were provided by the laboratory of the CDC. DNA was isolated from whole blood using the Puregene (D-50K) kit protocol (Gentra Systems, Inc., Minneapolis, MN) and stored at -80°C. One milliliter of extraction buffer was added to 10^5^ -10^8^ cells in whole blood, then centrifuged for 10 min at 13,000 g at 4-25°C. There was 500 μL binding buffer added to the spin column, centrifuged at 12,000 g for 30 s. There was 10 to 200 μL TE buffer added and incubated at room temperature for 1 min. The buffer in the microcentrifuge tube contains the DNA. DNA concentrations were measured by running aliquots on 0.8% agarose gel and by reading absorbance at 260 nm with a spectrophotometer. LTL was measured relative to standard reference DNA (T/S ratio), which was evaluated with samples from the human diploid fibroblast cell line IMR90. Using real-time quantitative polymerase chain reaction (qPCR), the relative ratio of telomere repeat copy number to single-copy gene copy number (T/S ratio) was determined in the blood leukocytes. Purified DNA samples were diluted in 96-well microtiter source plates to ∼1.75 ng/µl in 10 mM Tris–HCl, 0.1 mM EDTA, pH 7.5 (TE–4, final volume 300 µl/well), heated to 95°C for 5 min in a thermal cycler, quick chilled by transfer to an ice/water bath for 5 min, centrifuged briefly at 730 g, sealed with adhesive aluminum foil, and stored at 4°C until the time of assay. For each individual in whom the T/S ratio was assayed, three identical 20-µl aliquots of the DNA sample (35 ng/aliquot) were added to plate 1 and another three aliquots were added to the same well positions in plate 2. For each standard curve, one reference DNA sample was diluted serially in TE–4 by ∼1.68-fold per dilution to produce five concentrations of DNA ranging from 0.63 to 5 ng/µl, which were then distributed in 20-µl aliquots to the standard curve wells on each plate. The final concentrations of reagents in the PCR were 150 nM 6-ROX and 0.2× Sybr Green I (Molecular Probes), 15 mM Tris–HCl pH 8.0, 50 mM KCl, 2 mM MgCl2, 0.2 mM each dNTP, 5 mM DTT, 1% DMSO, and 1.25 U AmpliTaq Gold DNA polymerase (Applied Biosystems). The final telomere primer concentrations were: tel 1, 270 nM; tel 2, 900 nM. To standardize the variability between the two tests, each panel included eight control DNA samples. The sample characteristics were unknown to the LTL assay lab. The coefficient of variance between assays was 6.5%. The equation for conversion from the T/S ratio to bp pairs is 3274 + 2413 × (T/S) (30). The full details of this standard protocol have been described elsewhere (30–32). The CDC reviewed the quality of TL measurement before releasing the data to the public.

### Statistical Analysis and Sensitivity Analysis

Sample weights were used for analyses to account for the complex survey design and non-response of NHANES (30). Continuous and categorical variables were expressed as means ± standard deviation (SD) or proportions and were compared using weighted linear regression model or weighted χ2 test according to different TyG index groups (tertiles), respectively. The distribution of LTL levels according to sex, age, smoking status, and BMI were also described. The association between the TyG index and LTL was examined as a continuous variable per one unite increase and also as a categorical variable using tertiles with tertile 1 (T1) as the reference group. Multivariate linear regression analysis was applied to obtain beta coefficient (β) and 95% confidence interval (CI) for the association of the TyG index with LTL. Three models were constructed: model 1, adjusted for age; model 2, adjusted for age, sex, education, smoking status, alcohol consumption, physical activity, poverty to income ratio, and BMI; model 3, adjusted for age, sex, education, smoking status, alcohol consumption, physical activity, BMI, waist circumference, SBP, DBP, history of hypertension, TC, SUA, eGFR, and CRP. Variables considered as confounders based on existing literature, clinical judgment, and statistical significance in the univariate analysis were included. We further explored a dose-response relation between the TyG index by a decile approach [≤ 7.81 (reference), 7.81-8.04, 8.04-8.21, 8.21-8.37, 8.37-8.52, 8.52-8.68, 8.68-8.83, 8.83-9.03, 9.03-9.33, and >9.33] and LTL.

We conducted the sensitivity analysis to confirm the accuracy of our data analysis. If covariates with missing data were omitted from data analyses, we imputed missing values using the reference category for qualitative variables or the median value for quantitative variables. The imputed datasets in **Supplementary Table S1** were also used to get the main results. Second, the effects of the following variables on the relationship between the TyG index and LTL were investigated: sex (women vs. men), age (<45 vs. ≥45 years), BMI (<25 vs. ≥25 kg/m^2^), current smoking (yes vs. no), education (<high school vs. ≥high school), physical activity (sedentary, low, moderate vs. high), hypertension (yes vs. no), total cholesterol (<202 [median] vs. ≥202 mg/dL), and eGFR (<60 vs. ≥60 mL/min per 1.73 m^2^). Potential interactions were examined by including the interaction terms into those linear regression models with the greatest number of confounding variables.

All *P* values were two-sided with a significance level of < 0.05. Statistical analyses were performed using the statistical package R (http://www.R-project.org, The R Foundation) and Empower (R) (www.empowerstats.com; X&Y Solutions, Inc., Boston, MA). The figures were drawn using origin.

## Results

### Baseline Characteristics of Study Participants

A total of 6489 adults without diabetes were included in our study, with a mean age of 46.67 ± 18.55 years old. There were 3060 (47.16%) participants that were men. The mean (SD) TyG index was 8.6 (0.6) (median 8.5). The mean (SD) LTL was 5796.1 (691.4) bp (median 5722.3 bp). The weighted characteristics of the participants by TyG index tertiles are presented in **Table 1**. There were significant differences between TyG index tertiles. Participants with the highest TyG index in T3 (≥ 8.77) were more likely to be older, to be men, to be current smokers, to have lower educational levels and physical activity levels, to have a high rate of hypertension, to have higher values in BMI, waist circumference, SBP, DBP, FBG, TC, TG, SUA, and CRP, and to have lower values in poverty to income ratio, alcohol consumption, eGFR, and LTL than those of the other groups (all *P* < 0.01).

**Table 1:** Weighted characteristics of study population according to tertiles of the TyG index.

Variables^*^	Total participants	Tertiles of the TyG index			*P* value^¶^
		Tertile 1 (<8.27)	Tertile 2 (8.27-8.77)	Tertile 3 (≥ 8.77)	
N^†^	6489	2160	2166	2163	
Men, %	48	40.6	48.2	56.3	<0.001
Age, years	44.0 ± 16.3	39.8 ± 14.8	45.6 ± 17.1	47.2 ± 16.2	<0.001
BMI, kg/m^2‡^	27.7 ± 6.0	25.4 ± 5.3	28.0 ± 6.2	29.9 ± 5.9	<0.001
Waist circumference, cm	94.7 ± 15.1	87.5 ± 13.0	95.8 ± 14.9	101.9 ± 13.8	<0.001
SBP, mm Hg	117.5 ± 13.9	114.3 ± 12.8	118.3 ± 14.3	120.7 ± 14.0	<0.001
DBP, mm Hg	71.8 ± 11.7	70.2 ± 10.8	72.3 ± 11.8	73.3 ± 12.3	<0.001
Self-reported hypertension, %	21.9	13.2	23.9	29.6	<0.001
Poverty to income ratio	3.0 ± 1.6	3.1 ± 1.7	3.0 ± 1.6	2.9 ± 1.6	0.006
**Education, %**					<0.001
< High school	20.3	17.8	19.8	23.8	
High school	25.8	23.5	27.3	26.9	
> High school	53.9	58.7	52.9	49.4	
**Smoking status, %**					<0.001
Never	50.9	54.6	50.8	46.8	
Former	23.8	20.2	25.4	26.4	
Current	25.3	25.2	23.8	26.7	
Alcohol consumption, gm/day	12.3 ± 38.4	14.5 ± 43.9	10.6 ± 33.8	11.5 ± 36.0	0.002
**Physical Activity, %** ^§^					<0.001
Sedentary	20.2	16.4	21.7	22.9	
Low	27.4	25.3	28.2	29.0	
Moderate	19.6	21.6	18.7	18.1	
High	32.9	36.8	31.3	30.0	
**Laboratory examination**					
FBG, mg/dL	89.2 ± 12.4	85.7 ± 8.2	88.8 ± 8.5	93.8 ± 17.4	<0.001
TC, mg/dL	203.0 ± 40.6	184.7 ± 34.8	204.2 ± 37.0	222.6 ± 40.9	<0.001
TG, mg/dL	136.3 ± 101.8	66.3 ± 15.1	115.1 ± 19.4	238.1 ± 126.0	<0.001
TyG index	8.5 ± 0.6	7.9 ± 0.2	8.5 ± 0.1	9.2 ± 0.4	<0.001
SUA, mg/dL	5.3 ± 1.5	4.8 ± 1.3	5.3 ± 1.4	5.9 ± 1.4	<0.001
eGFR, mL/min per 1.73 m^2^	102.5 ± 22.6	107.0 ± 21.6	101.4 ± 22.8	98.6 ± 22.7	<0.001
CRP, mg/dL	0.4 ± 0.7	0.3 ± 0.7	0.4 ± 0.7	0.5 ± 0.7	<0.001
Telomere length, T/S ratio	1.1 ± 0.3	1.1 ± 0.3	1.1 ± 0.3	1.0 ± 0.3	<0.001
Telomere length, bp	5858.2 ± 675.1	5962.8 ± 665.4	5836.7 ± 650.7	5761.4 ± 694.5	<0.001

BMI, body mass index; SBP, systolic blood pressure; DBP, diastolic blood pressure; FBG, fasting blood glucose; TC, total cholesterol; TG, triglycerides; TyG, triglyceride glucose; SUA, serum uric acid; eGFR, estimated glomerular filtration rate; CRP, C-reactive protein; LTL, leukocyte telomere length.^*^Mean ± SD for continuous variables and percentages for categorical variables were weighted. ^¶^P values of continuous variables and categorical variables were calculated by weighted linear regression model and weighted chi-square test, respectively.^†^Unweighted sample number in the dataset.^‡^BMI was calculated as the body weight in kilograms divided by the square of the height in meters.^§^The Physical Activity categories were based on the distribution of MET-minute levels for the present NHANES sample.

**Figure 2:**
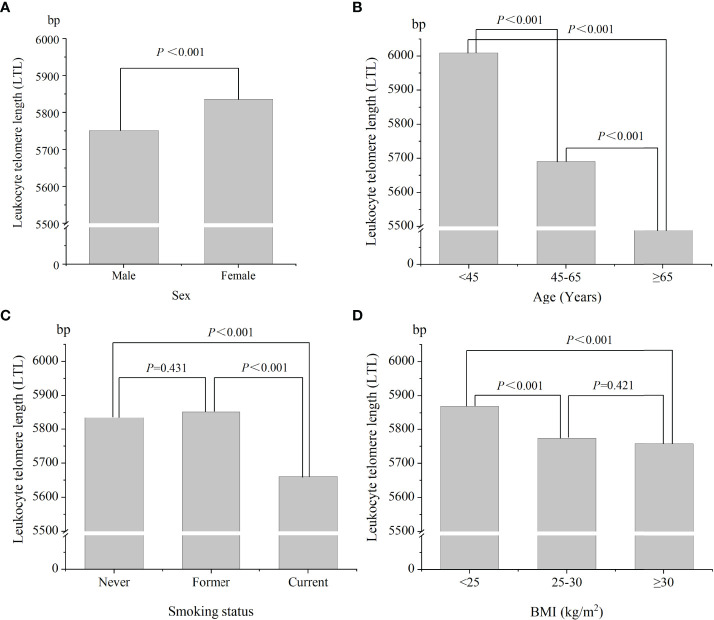
The distributions of leukocyte telomere length in different population characteristics. **(A)** Sex; **(B)** age; **(C)** smoking status; **(D)** BMI.

**Figure 2** shows the distribution of LTL levels according to sex, age, smoking status, and BMI. As shown in **Figure 2A**, women had higher LTL levels than that in men (5836.1 vs 5751.3 bp, *P* < 0.01). Participants with increasing level of age had lower LTL values (*P* < 0.001) (**Figure 2B**). The mean LTL values in never smoker, former smoker, and current smoker were 5834.9 ± 736.7, 5852.3 ± 640.0, and 5660.6 ± 617.2 bp, respectively (**Figure 2C**). Current smoker had lower LTL levels than that in other groups. There was no statistical difference in LTL levels between never smoker and former smoker (*P* = 0.431). The mean LTL values for BMI of < 25, 25-30, and ≥ 30 kg/m^2^ were 5868.9 ± 801.2, 5773.8 ± 630.6, and 5758.1 ± 616.3 bp, respectively (**Figure 2D**). Participants with normal weight had higher LTL levels than that in overweight/obese subjects (*P* < 0.001). There was no statistical difference in LTL levels between overweight and obese people (*P* = 0.421).

### Variables Associated With LTL Levels

The associations between various baseline characteristics and LTL levels using univariate analysis are shown in **Supplementary Table S1**. The LTL levels were significantly associated with sex, BMI, waist circumference, SBP, DBP, history of hypertension, education levels, smoking status, alcohol consumption, physical activity, TC, CRP, SUA, eGFR, and the TyG index, but not poverty to income ratio.

### Association Between TyG Index and LTL Levels

The association between the TyG index and LTL in the absence of diabetes using multivariate linear regression analyses is further illustrated in **Table 2**. We constructed 3 models for analyzing the role of the TyG index in LTL levels. After adjustment for the important confounders, the positive association between the TyG index and LTL was still found in all models. In model 3, for each 1 unit increase in the TyG index, the change in LTL was -72.62 bp (95%CI: -107.36~-37.87). The TyG index was converted from a continuous variable to a categorical variable (tertiles). Compared with those with TyG index < 8.27, subjects with TyG index ≥ 8.77 had statistically significantly shorter LTL (β = -95.98, 95%CI: -145.08~-46.89, *P*<0.001), while no association was found between middle TyG index tertile (8.27-8.77) and LTL (β = -4.31, 95%CI: -48.12~39.49, *P*=0.847). Similarly, a significantly shorter LTL (β = -93.33, 95%CI: -134.33~-52.32) was found in participants in tertile 3 (≥ 8.77) compared with participants in tertiles 1-2 (< 8.77). The results did not change qualitatively when we reanalyzed the association between TyG index and LTL using imputation data (**Supplementary Table S2**). It seems that the association between the TyG index and LTL is likely to be nonlinear. TyG index ≥ 8.77 was inversely associated with LTL, supporting a threshold effect of the TyG index on LTL among U.S. adults without diabetes.

**Table 2:** Association of the TyG index with leukocyte telomere length.

TyG index	LTL, bp					
	Model 1		Model 2		Model 3	
	β (95% CI)	*P* value	β (95% CI)	*P* value	β (95% CI)	*P* value
Continuous	-61.95 (-88.23, -35.66)	<0.001	-58.87 (-86.28, -31.47)	<0.001	-72.62 (-107.36, -37.87)	<0.001
Tertiles						
T1 (<8.27)	0 (Reference)		0 (Reference)		0 (Reference)	
T2 (8.27-8.77)	-27.07 (-65.46, 11.32)	0.167	-7.75 (-46.27, 30.78)	0.694	-4.31 (-48.12, 39.49)	0.847
T3 (≥ 8.77)	-87.77 (-126.34, -49.21)	<0.001	-78.50 (-118.25, -38.75)	0.003	-95.98 (-145.08, -46.89)	<0.001
Categories						
T1-T2 (<8.77)	0 (Reference)		0 (Reference)		0 (Reference)	
T3 (≥ 8.77)	-73.86 (-107.00, -40.72)	<0.001	-74.24 (-107.87, -40.61)	<0.001	-93.33 (-134.33, -52.32)	<0.001

TyG, triglyceride glucose; LTL, leukocyte telomere length; CI, confidence interval. Model 1 was adjusted for age. Model 2 was adjusted for age, sex, education, smoking status, alcohol consumption, physical activity, and BMI. Model 3 was adjusted for age, sex, education, smoking status, alcohol consumption, physical activity, BMI, waist circumference, SBP, DBP, history of hypertension, TC, SUA, eGFR, and CRP.

**Figure 3:**
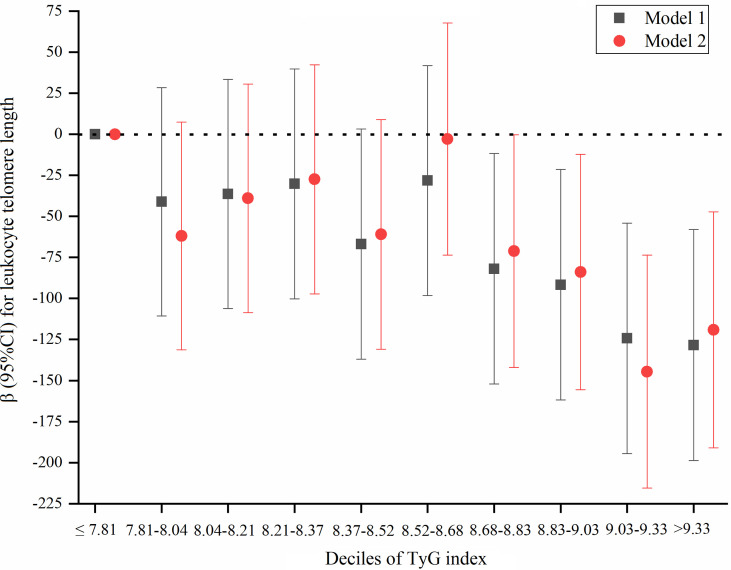
A dose-response association between TyG index by a decile approach and LTL. Model 1 was adjusted for age; model 2 was adjusted for age, sex, education, smoking status, alcohol consumption, physical activity, BMI, waist circumference, SBP, DBP, history of hypertension, TC, SUA, eGFR, and CR"P".

We further explored a dose-response relation between the TyG index by a decile approach [≤ 7.81 (reference), 7.81-8.04, 8.04-8.21, 8.21-8.37, 8.37-8.52, 8.52-8.68, 8.68-8.83, 8.83-9.03, 9.03-9.33, and >9.33] and LTL (**Figure 3**). We constructed 2 models: model 1, only adjusted for age; model 2, adjusted for age, sex, education, smoking status, alcohol consumption, physical activity, BMI, waist circumference, SBP, DBP, history of hypertension, TC, SUA, eGFR, and CRP. Compared to TyG index ≤ 7.81, 5 subgroups (TyG index 7.81-8.04, 8.04-8.21, 8.21-8.37, 8.37-8.52, and 8.52-8.68) did not show significant effect on LTL, while there was a significantly shorter LTL for participants with TyG index > 8.68. The results also suggest that there was a threshold effect of the TyG index on LTL.

We further analyzed the association of HOMA-IR with LTL. As shown in **Supplementary Table 3S**, after adjustment for important confounders, HOMA-IR was not associated with LTL, suggesting that the TyG index was better associated with LTL than HOMA-IR.

## Discussion

We demonstrated that high TyG index levels (> 8.68) were associated with shorter LTL among U.S. adults without diabetes, compared to low TyG index levels, supporting a threshold effect of the TyG index on LTL, while HOMA-IR was not. The results suggest that abnormal glucose metabolism might be associated with biological age, oxidative stress, and inflammation of blood vessels. Our findings, if further confirmed, have potential clinical implications.

Of note, serval studies have investigated the IR-LTL association, and the results are also conflicting. There was an inverse association between HOMA-IR, LTL, and LTL in a cross-sectional study of 303 adults free of cardiovascular disease (16). Al-Attas et al. used data from 193 middle-aged Arabs and found that HOMA-IR was inversely associated with LTL in men (33). A recent observation study included 305 participants with type 2 diabetes demonstrated that both telomere length and telomerase activity progressively decreased as IR increased (34). GEMINAKAR study conducted in 338 (184 monozygotic and 154 dizygotic) same-sex twin pairs and failed to find a significant IR-LTL association (35). However, IR is difficult to be widely used in the clinic because it is time-consuming, expensive, and there is a complexity of detection methods (10). Also, there is a particular lack of data on the role of the TyG index on LTL in the non-diabetic population. Guerrero-Romero et al. suggested that the TyG index was more sensitive and specific than the normal blood glucose hyperinsulinemic clamp test, indicating that the TyG index could be used to identify subjects with decreased insulin sensitivity, regardless of whether the subjects had type 2 diabetes (10). It was found that the TyG index might be a useful independent predictor for coronary artery calcification progression in 1175 adult Koreans (36). Our result indicated that the TyG index, but not HOMA-IR, was independently and inversely associated with LTL in healthy adults and observed a threshold effect of the TyG index on LTL. High TyG index levels (> 8.68) were associated with shorter LTL among U.S. adults without diabetes, compared to low TyG index levels. As far as we know, the “optimal level” of the TyG index in non-diabetic people has yet to be determined. Our study found that the possible optimal levels of the TyG index might be 8.68 with respect to LTL. Compared with previously reported studies, our study was the first report to explore the association between LTL and two simple markers of IR, that is, HOMA-IR and the TyG index and to assess the nonlinear relationship between the TyG index and LTL in U.S. adults free of diabetes. We handled the target independent variable as both a continuous variable and as a categorical variable. Such an approach can reduce the contingency in the data analysis and enhance the robustness of results. Additionally, our study included larger sample sizes, which caused the standard error to be smaller, thus improving the test performance. Our findings suggest that the TyG index was better associated with LTL than HOMA-IR and the TyG index might be expected to be an independent predictor of metabolic diseases which is of great significance for disease treatment and monitoring, while it warrants further investigation due to the unclear mechanism between LTL and IR.

There were several possible reasons that might account for the association between high TyG index and shorter LTL. A high level of the TyG index means that there is a high IR and glycometabolism disorder. When long-term at high glucose levels, reactive oxygen species (ROS), mitochondrial dysfunction, and inflammatory response were generated and accumulated, which can directly and indirectly attack and damage DNA and telomerase (37, 38). Meanwhile, the excessive serum TG may lead to the accumulation of fatty acids in non-adipose cells resulting in lipotoxicity and lipoapoptosis along with damaging the integrity and proliferation of cellular genetic material (39, 40). Although telomere shortening in leucocytes is widely interpreted in relation to cell ageing (41), it is also a real forerunner for a comprehensive assessment of the cumulative lifelong burden of oxidative stress and inflammation in the current metabolic status (18). Demissie et al. observed that hypertension augmented insulin resistance and oxidative stress were associated with shorter LTL, and that the level of LTL in hypertensives is largely due to insulin resistance (18). This could also tie in with activation in adipose tissue and its link with subclinical chronic inflammation (42, 43). Moreover, the association between IR and LTL could be due to higher cell damage and turnover, which accelerates cell ageing by pushing cells to their maximal replicative capacity, resulting in shorter LTL. Our findings suggest that IR may increase the susceptibility to disease by shortening the length of telomerase in healthy adults.

It is also worth noting that our study has several limitations. First, because of the cross-sectional nature of the data, our findings do not contribute to an understanding of the directionality of any correlations. No causal inference can be drawn from our findings. Second, because those participants with incomplete data were omitted from modified models, selection bias may have occurred. However, we employed multiple imputation for missing covariates to reduce bias. Third, the study participants were all U.S. civilians. Thus, the results’ generalizability to other populations remains to be confirmed. Despite these limitations, our research yielded benefits. First, our study was the first report to demonstrate that the TyG index, but not HOMA-IR, was associated with LTL in U.S. adults without diabetes. Second, we found a nonlinear association between the TyG index and LTL. Third, we presented adequate statistical explanation for the assessment of independent risk from the TyG index, a feature that was lacking in previous studies.

## Conclusions

In conclusion, high TyG index (>8.68), not HOMA-IR, was inversely and independently associated with LTL among non-diabetic U.S. adults. Our data suggest the broad clinical applicability of the TyG index early on as a prognostic indicator. It warrants further investigation.

## Data Availability Statement

The datasets presented in this study can be found in online repositories. The names of the repository/repositories and accession number(s) can be found below: The datasets are available on DataDryad (https://doi.org/10.5061/dryad.d5h62).

## Ethics Statement

NHANES study protocols were approved by the research ethics review board of the National Center for Health Statistics. The patients/participants provided their written informed consent to participate in this study.

## Author Contributions

GH and JL conceived and designed the research; LH, QZ, YB, and GH participated in acquisition of data, or analysis and interpretation of data; LH wrote the original draft manuscript; QZ, YB, GH, and JL reviewed and edited the manuscript; LH, GH, and JL were involved in funding acquisition. All the authors approved the final version of the manuscript and agree to be accountable for all aspects of the work.

## Funding

The study was supported by the National Key Research and Development Program of China (grant numbers 2021YFC2500600, 2021YFC2500601); Peking University Medicine Fund of Fostering Young Scholars’ Scientific & Technological Innovation (grant numbers 34254); Open Fund for Key Laboratory of Chemical Pollution and Health and Safety of China Center for Disease Control and Prevention (grant numbers 2020CDCKL02), top-notch personnel program of Beihang University (grant numbers YWF-20-BJ-J-1053) and the National Natural Science Foundation of China (grant numbers 82003427).

## Conflict of Interest

The authors declare that the research was conducted in the absence of any commercial or financial relationships that could be construed as a potential conflict of interest.

## Publisher’s Note

All claims expressed in this article are solely those of the authors and do not necessarily represent those of their affiliated organizations, or those of the publisher, the editors and the reviewers. Any product that may be evaluated in this article, or claim that may be made by its manufacturer, is not guaranteed or endorsed by the publisher.

## Abbreviations

LTL, leukocyte telomere length; IR, insulin resistance; TyG index, triglyceride-glucose index; HOMA-IR, homeostasis model assessment of IR; CVD, cardiovascular disease; PCR, polymerase chain reaction; GAM, generalized additive model; BMI, body mass index; SBP, systolic blood pressure; DBP, diastolic blood pressure; FBG, fasting blood glucose; TC, total cholesterol; TG, triglycerides; TyG, triglyceride glucose; SUA, serum uric acid; eGFR, estimated glomerular filtration rate; CRP, C-reactive protein.

**Supplementary Table S1**. Univariate analysis for leukocyte telomere length.

**Supplementary Table S2**. Association of TyG index with leukocyte telomere length among pro-imputation data.

**Supplementary Table S3**. Association of HOMA-IR with leukocyte telomere length.
