# Supplementary material for: Triglyceride-Glucose Index Correlate With Telomere Length in Healthy Adults From the National Health and Nutrition Examination Survey
Source: Front Endocrinol (Lausanne). 2022 Jun 2;13:844073. doi: 10.3389/fendo.2022.844073 (PMC9201959; doi:10.3389/fendo.2022.844073)
Supplement: Supplementary file 1 [file Table_1.docx]

Supplementary Material

**Table S1. Univariate analysis for leukocyte telomere length**

| Covariate | β (95% CI) | *P* value |
| --- | --- | --- |
| Age, years | -14.24 (-15.08, -13.40) | <0.001 |
| Sex |  |  |
| Female | 0 |  |
| Male | -84.83 (-118.47, -51.19) | <0.001 |
| BMI, kg/m^2^ | -5.45 (-8.27, -2.62) | <0.001 |
| Waist circumference, cm | -5.66 (-6.82, -4.51) | <0.001 |
| SBP, mm Hg | -5.85 (-7.11, -4.59) | <0.001 |
| DBP, mm Hg | -1.52 (-2.98, -0.07) | 0.040 |
| Self-reported hypertension |  |  |
| No | 0 |  |
| Yes | -176.11 (-214.89, -137.32) | <0.001 |
| Poverty to income ratio | 5.03 (-5.88, 15.95) | 0.366 |
| **Education** |  |  |
| < high school | 0 |  |
| High school | 115.34 (69.64, 161.03) | <0.001 |
| > high school | 145.46 (106.62, 184.29) | <0.001 |
| **Smoking status** |  |  |
| Never | 0 |  |
| Former | -174.35 (-215.26, -133.44) | <0.001 |
| Current | 17.40 (-24.60, 59.39) | 0.417 |
| Alcohol consumption, gm/day | 0.51 (0.05, 0.96) | 0.028 |
| **Physical Activity** |  |  |
| Sedentary | 0 |  |
| Low | 99.53 (51.96, 147.11) | <0.001 |
| Moderate | 85.57 (31.83, 139.30) | 0.002 |
| High | 142.22 (95.15, 189.28) | <0.001 |
| TC, mg/dL | -1.67 (-2.08, -1.27) | <0.001 |
| CRP (mg/dL) | -59.12 (-79.46, -38.77) | <0.001 |
| SUA, mg/dL | -44.33 (-55.50, -33.16) | <0.001 |
| eGFR, mL/min per 1.73 m^2^ | 6.80 (6.16, 7.44) | <0.001 |
| TyG index | -136.78 (-164.64, -108.93) | <0.001 |

Abbreviations: BMI, body mass index; SBP, systolic blood pressure; DBP, diastolic blood pressure; TC, total cholesterol; TyG, triglyceride glucose; SUA, serum uric acid; eGFR, estimated glomerular filtration rate; CRP, C-reactive protein; CI, confidence interval.

**Table S2. Association of TyG index with leukocyte telomere length among proimputation data.**

| TyG index | LTL, bp | | | |
| --- | --- | --- | --- | --- |
|  | Model 1 | | Model 2 | |
|  | β (95% CI) | *P* value | β (95% CI) | *P* value |
| Continuous | -61.95 (-88.23, -35.66) | <0.001 | -34.12 (-64.88, -3.37) | 0.030 |
| Tertiles |  |  |  |  |
| T1 (<8.27) | 0 (Reference) |  | 0 (Reference) |  |
| T2 (8.27-8.77) | -27.07 (-65.46, 11.32) | 0.167 | -2.16 (-42.06, 37.74) | 0.916 |
| T3 (≥ 8.77) | -87.77 (-126.34, -49.21) | <0.001 | -47.55 (-91.50, -3.60) | 0.034 |
| Categories |  |  |  |  |
| T1-T2 (<8.77) | 0 (Reference) |  | 0 (Reference) |  |
| T3 (≥ 8.77) | -73.86 (-107.00, -40.72) | <0.001 | -46.20 (-82.41, -9.99) | 0.012 |

Abbreviations: TyG, triglyceride glucose; LTL, leukocyte telomere length; CI, confidence interval.

Model 1 was adjusted for age.

Model 2 was adjusted for age, sex; education, smoking status, alcohol consumption, physical activity, BMI, waist circumference, SBP, DBP, history of hypertension, TC, SUA, eGFR and CRP.

**Table S3. Association of HOMA-IR with leukocyte telomere length.**

| HOMA-IR | LTL, bp | | | | | |
| --- | --- | --- | --- | --- | --- | --- |
|  | Model 1 | | Model 2 | | Model 3 | |
|  | β (95% CI) | *P* value | β (95% CI) | *P* value | β (95% CI) | *P* value |
| Continuous | -12.85 (-21.56, -4.14) | 0.004 | -4.59 (-14.85, 5.67) | 0.380 | -2.85 (-15.01, 9.30) | 0.646 |
| Tertiles |  |  |  |  |  |  |
| T1 | 0 (Reference) |  | 0 (Reference) |  | 0 (Reference) |  |
| T2 | -43.64 (-90.09, 2.81) | 0.066 | -29.65 (-81.22, 21.92) | 0.260 | -26.48 (-86.13, 33.18) | 0.385 |
| T3 | -91.64 (-140.34, -42.94) | <0.001 | -50.61 (-111.99, 10.77) | 0.106 | -51.90 (-124.06, 20.25) | 0.159 |
| *P* for trend | <0.001 |  | 0.100 |  | 0.155 |  |

Abbreviations: HOMA-IR, homeostatic model assessment of insulin resistance; LTL, leukocyte telomere length; CI, confidence interval.

Model 1 was adjusted for age.
Model 2 was adjusted for age, sex; education, smoking status, alcohol consumption, physical activity and BMI.

Model 3 was adjusted for age, sex; education, smoking status, alcohol consumption, physical activity, BMI, waist circumference, SBP, DBP, history of hypertension, TC, SUA, eGFR and CRP.
